# The In-Situ Synthesis of a 3D SnS/N-Doped Graphene Composite with Enhanced Electrochemical Performance as a Low-Cost Anode Material in Sodium Ion Batteries

**DOI:** 10.3390/ma12122030

**Published:** 2019-06-25

**Authors:** Ning-Jing Song, Canliang Ma

**Affiliations:** 1 College of Chemistry and Chemical Engineering, Jinzhong University, Jinzhong 030619, China; snj642370134@126.com; 2 Key Laboratory of Materials for Energy Conversion and Storage of Shanxi Province, Institute of Molecular Science, Shanxi University, Taiyuan 030006, China

**Keywords:** SnS/N-doped graphene, sodium ion battery, in-situ synthesis, phase transformation

## Abstract

SnS/N-doped graphene (SnS/NG) composites are promising anode materials for sodium ion batteries. Generally, SnS is synthesized from SnCl_2_·2H_2_O. However, SnCl_2_·2H_2_O is not suitable for large-scale production due to its high price. Compared with SnCl_2_·2H_2_O, SnCl_4_·5H_2_O has a lower price, more stable chemical properties and better water solubility. Until now, there have been no related reports on the synthesis of SnS from SnCl_4_·5H_2_O. In this work, the fabrication of SnS/NG in a facile, two-step process, which combines a hot water bath and thermal annealing and uses SnCl_4_·5H_2_O as a precursor, is described. The mechanism of phase transformation in the direct synthesis of SnS from Sn^4+^ is also discussed in detail. Applying our methodology, SnS nanoparticles were grown in-situ on graphene sheets and wrapped by N-doped graphene sheets to form a 3D SnS/NG composite. With 35.35% content of graphene in the SnS/NG composite, the reversible specific capacity remained at 417.8 mAh/g at 1000 mA/g after 100 cycles, exhibiting a high specific capacity and good cycling stability. In addition, the composite also had an excellent rate performance, with a specific capacity of 366.9 mAh/g obtained even at 5000 mA/g. Meanwhile, the fast sodium storage kinetics of SnS/NG were also analyzed, providing some theoretical support for further study.

## 1. Introduction

Motivated by sodium’s similar physicochemical properties to lithium, abundance and low-cost, sodium ion batteries (SIBs) hold promise for a broad range of energy storage applications in the future [1]. Developing high-performance anode and cathode materials for SIBs is one of the most popular research subjects. Based on the theoretical stoichiometry of Na_15_Sn_4_ (847 mAh/g)—as SIBs anode—tin sulfide compounds are considered one type of promising material [2,3,4]. 

Owing to their large layer spacing (5.90 Å for SnS_2_ and 4.33 Å for SnS vs 1.02 Å for Na^+^) and high theoretical specific capacity, SnS_2_ and SnS have been intensively explored as anode materials for SIBs [5,6,7]. Compared with SnS_2_, SnS exhibits a smaller volume change (242% vs. 324% for SnS_2_) and less phase transformation upon sodiation/desodiation, which makes it more suited for repeated cycling operations—even though its intrinsic energy capacity is relatively low [8]. Zhou et al. [8] found that SnS_2_ could transform into SnS after an annealing step in an argon atmosphere, and that the transformed SnS showed enhanced sodium ion storage performance compared to that of SnS_2_. Currently, the regular synthesis method of SnS involves a hot water bath or hydrothermal reaction with SnCl_2_·2H_2_O and thioacetamide (TAA) as precursors. Compared with SnCl_2_·2H_2_O, SnCl_4_·5H_2_O has a lower price, more stable chemical properties and better water solubility. Thus, from the perspective of reducing the cost of electrode materials, it would be a better selection to synthesize SnS nanoparticles using SnCl_4_·5H_2_O as the precursor in aqueous solution. Up to now, there have been no reports about the synthesis of SnS using SnCl_4_·5H_2_O as a precursor.

In order to solve the rapid capacity fading, poor rate performance, serious agglomeration and volume expansion of pure SnS, application of SnS/carbon composites has been a common remedy as they can increase electrical conductivity, prevent the aggregation of nanoparticles and accommodate volume change [9,10]. Owing to its exceptional electrical, mechanical and chemical properties, graphene is often used as carrier for SnS. Based on this, the electrical conductivity and cycle stability of SnS/G composites has been shown to increase significantly [5,11]. However, strong Van der Waals forces and weak bonding between nonpolar graphene and polar tin sulfide can lead to the restacking of graphene and agglomeration of tin sulfide during repeated sodiation/desodiation processes. Different from pure graphene, N-doped graphene (NG) may offer a stronger affinity to active materials and is easier to assemble into 3D structures with less restacking. Xiong et al. [7] prepared 3D SnS/NG by selecting commercial SnS_2_ and (NH_4_)_2_S as precursors and adding Poly (diallyldimethylammonium chloride)-graphene oxide (PDDA-GO) solution to the above solution, using dicyandiamide as a nitrogen source. Their 3D SnS/NG achieved better cycling performance than that of SnS/G. 

In order to further reduce the cost of electrode materials and process complexity, we selected low-cost and water soluble SnCl_4_·5H_2_O and TAA as precursors and common NH_3_·H_2_O as a nitrogen source to prepare the 3D SnS/NG composite via a simple and controllable two-step method, which combined a hot water bath and thermal annealing. During the process, in-situ synthesis of SnS and N-doping of graphene were realized simultaneously. As a result, SnS nanoparticles were anchored on the N-doped graphene sheets and wrapped by them. For SnS/NG, SnS offers high specific capacity, while graphene supplies excellent electrical conductivity. Meanwhile, owing to the in-situ synthesis and N-doping of graphene, agglomeration was prevented and volume changes were alleviated significantly. Thus, SnS/NG possessed a high reversible capacity, an excellent rate capability and a stable cyclic performance, showing great potential as an anode material for SIBs. In this paper, the following three problems are discussed in detail: (1) The reaction mechanism from Sn^4+^ to SnS in the whole process, (2) the cycling stability and rate performance of SnS, SnS/G and SnS/NG as anode materials for SIBs, and (3) the fast sodium storage kinetics of SnS/NG. 

## 2. Materials and Methods

A graphene oxide suspension (1.5 mg/mL) was prepared by sonicating a GO dispersion for 2 h. Then, 0.601 g thioacetamide (TAA), 0.702 g SnCl_4_·5H_2_O and 25 mg polyvinyl pyrrolidone (PVP) was added to 40 mL of the graphene oxide suspension under sonication for 30 min. Then, 20 mL of NH_3_·H_2_O was added to the mixed solution before it was heated at 90 °C for 1 h. After the solution was allowed to naturally cool to an ambient temperature, the product was collected by centrifugation and freeze-dried overnight. Finally, the obtained materials were heated to 600 °C at a rate of 5 °C/min in an Ar atmosphere, and kept at 600 °C for 120 min. The obtained hybrid was marked as SnS/NG. In addition, SnS/G was prepared without adding NH_3_·H_2_O, while pure SnS nanoparticles were prepared without adding NH_3_·H_2_O or graphene.

The as-obtained samples were characterized using a scanning electron microscope (SEM, JSM-7001F, 3.0 kV, Japan), an X-ray diffractometer (XRD, Cu Ka radiation, D8 Advance, BRUKER/AXS, Germany), and an X-ray photoelectron spectrometer (XPS, Thermo ESCALAB 250XI, Thermo Fisher Scientific, USA). The nitrogen adsorption/desorption isotherms were recorded by a Micromeritics ASAP 2010 surface area analyzer. Thermo-gravimetric analysis (TGA, STA 1640, Stanton Redcroft Inc., UK) was performed at a heating rate of 10 °C min^−1^ in air. The electrochemical properties were measured using coin-type (CR2032) half cells. To produce a slurry, 80 wt.% samples, 10 wt.% acetylene black and 10 wt.% carboxymethyl cellulose were mixed in deionized water. This slurry was then uniformly loaded on a Cu foil with a doctor blade to prepare a film-type electrode. The sample was dried at 100 °C under vacuum for 12 h, and then cut into circular electrodes. The cells were assembled in an Ar-filled glove box (Dellix Co., Chengdu, China) with sodium foil as both the reference and counter electrode, glass fiber as the separator and a solution of 1.0 M NaClO_4_ in ethylene carbonate (EC): dimethyl carbonate (DMC): ethyl methyl carbonate (EMC) = 1:1:1 vol.% with 2.0% fluoroethylene carbonate (FEC) additive as the electrolyte. All electrochemical measurements were carried out on a battery testing system (Neware Co., Shenzhen, China) in the potential range of 0.01 V to 3 V. Cyclic voltammetry (CV) measurements were made using an IM6 electrochemical testing station running at 0.1 mVs^−1^ from the open circuit potential to 0.01 V, and then back to 2.5 V.

## 3. Results and Discussion

**Figure 1:**
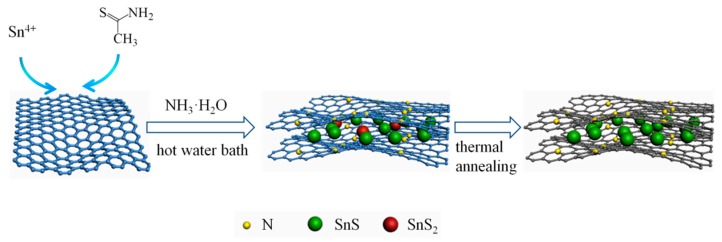
Schematic of synthesis of SnS/N-doped graphene (NG).

As shown in Figure 1, SnS/NG was prepared by a facile procedure involving a hot water bath and thermal annealing. Firstly, SnCl_4_·5H_2_O and TAA were dissolved in graphene oxide (GO) in order to prepare a suspension of mixed solution. During the hot water bath, as NH_3_·H_2_O was added to the above suspension, nitrogen atoms were doped into GO sheets, which provided more defect sites for the formation of tin sulfide compound particles. Meanwhile, Sn^4+^ was partly reduced to form SnS in the presence of NH_3_·H_2_O, while the remainder generated SnS_2_, as confirmed by the XRD results. Therefore, SnS and SnS_2_ were successfully grown in-situ on N-doped graphene oxide sheets. Secondly, during the process of high temperature thermal reduction, N-doped graphene oxide nanosheets were reduced to N-doped graphene nanosheets, while SnS_2_ was reduced to SnS. As a result, SnS was grown in-situ on N-doped graphene sheets, forming a 3D SnS/NG composite.

**Figure 2:**
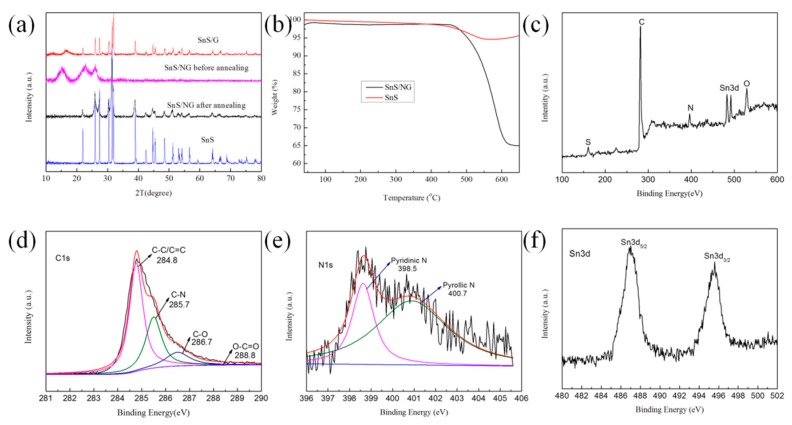
(**a**) X-ray diffraction (XRD) patterns of SnS, SnS/graphene (SnS/G), and SnS/NG before and after heat treatment; (**b**) thermo-gravimetric analysis (TGA) curve of the SnS/NG and SnS hybrid in air; (**c**) survey X-ray photoelectron spectrometer (XPS) spectra; and high-resolution XPS spectra of (**d**) C 1s, (**e**) N 1s and (**f**) Sn 3d of SnS/NG.

XRD was used to identify the phase compositions and structure of pure SnS, SnS/G, and SnS/NG before annealing and SnS/NG after annealing (Figure 2a). For SnS and SnS/NG after annealing, all of the high crystallinity peaks could be indexed to SnS (PDF#65-3812). The lattice distance of SnS is 2.84 Å, which is larger than the size of Na^+^ at 1.02 Å. Thus, the lattices were considered fit for hosting Na^+^. No apparent graphene diffraction peaks at 2θ = 20–30° were observed, which was in agreement with the findings of Reference [7]. For SnS/NG before annealing, the peaks located at 21.946° and 25.952° could be indexed as SnS (PDF#65-3812), meanwhile, the peak located at 15.029° could be indexed as SnS_2_ (PDF#23-0677), which was consistent with the results obtained by References [11] and [12]. A possible explanation for this phenomenon is that a portion of Sn^4+^ was reduced to Sn^2+^ during the hot water bath reaction—owing to the existence of NH_3_·H_2_O—while the rest of the Sn^4+^ was reduced to Sn^2+^ during the thermal reduction. For SnS/G, a peak located at 16.3° could be indexed as SnS_2_ (PDF#23-0677). Therefore, through comparison of SnS/G and SnS/NG after annealing, ammonia reduction and thermal annealing were the determining factors in the complete reduction of Sn^4+^ to Sn^2+^, and both reduction processes were indispensable.

The content of graphene among SnS/NG was estimated by TGA. As shown in Figure 2b, when the temperature reached 455 °C, the weight of SnS/NG decreased sharply. This could be attributed to a loss of graphene. In addition, the weight of SnS also decreased slightly, which could be due to the existence of a little PVP. The content of graphene in the SnS/NG was calculated as 30.35%. 

XPS was carried out to determine the state of the chemical bonds in the samples. As shown in Figure 2c, the survey XPS spectra of SnS/NG had a pronounced N1 peak at 400.0 eV; the content of N was found to be 5.37% by elemental analysis from the survey spectrum, implying that the graphene sheet was successfully doped with N. The content of O was 10.68% in SnS/NG, lower than pure graphene oxide, which could be attributed to the observed reduction of graphene oxide. High-resolution XPS spectra of C 1s was fitted into four peaks with binding energies (B.E.) at about 284.4, 285.7, 286.7 and 288.8 eV, attributable to C–C/C=C, C–N, C–O and O–C=O, respectively (Figure 2d). Our result was in agreement with that of Reference [7]. The content of C–C/C=C, C–N, C–O and O–C=O was 44.11%, 24.27%, 9.87% and 0.85%, respectively. From this, we found that N in SnS/NG combined with C to form C–N. As shown in Figure 2e, N 1s fitted into two peaks, indicating two kinds of N existed, namely pyridinic N (398.5 eV) and pyrrolic N (400.7 eV). In addition, the peak of Sn 3d_2/5_ was located at 486.9 eV, corresponding to SnS (Figure 2f), which was consistent with the result of the XRD spectrum. Thus, we were able to confirm that SnS/NG was successfully synthesized by the simple two-step method.

**Figure 3:**
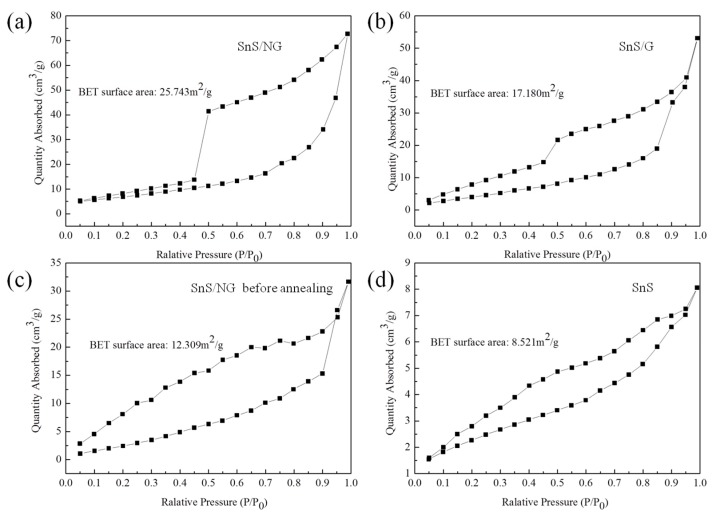
N_2_ isotherms and calculated Brunauer–Emmett–Teller (BET) specific surface area of (**a**) SnS/NG, (**b**) SnS/G, (**c**) SnS/NG before annealing and (**d**) SnS.

The N_2_ adsorption/desorption isotherms of SnS/NG, SnS/G, SnS/NG before annealing and SnS are shown in Figure 3. The Brunauer–Emmett–Teller (BET) surface area of SnS was 8.521 m^2^ g^−1^ (Figure 3d). With the addition of graphene, the BET surface area of SnS/NG, SnS/G and SnS/NG before annealing increased to 25.743 m^2^ g^−1^ (Figure 3a), 17.180 m^2^ g^−1^ (Figure 3b) and 12.309 m^2^ g^−1^ (Figure 3c), respectively; the major contributor to surface area was the graphene sheets. Compared to SnS/NG before annealing and SnS/G, SnS/NG had the highest specific surface area, which could be attributed to the removal of oxygen-containing functionality, N-doping of GO during the annealing process and a chemical reduction. This was consistent with our XPS results.

**Figure 4:**
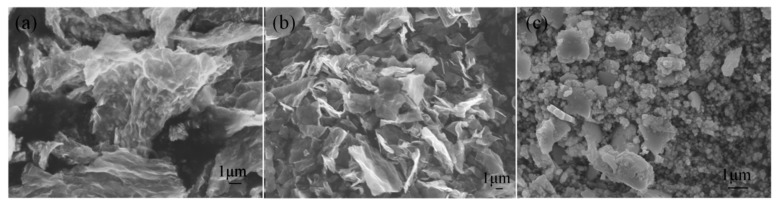
Scanning electron microscope (SEM) images of (**a**) SnS/NG, (**b**) SnS/G, and (**c**) SnS.

SEM images of SnS/NG, SnS/G and SnS are shown in Figure 4. As shown in Figure 4a, for SnS/NG, the SnS nanoparticles were wrapped by the graphene sheets and uniform in size. For SnS/G, the stacking of graphene sheets was more obvious (Figure 4b), while pure SnS nanoparticles exhibited dense agglomeration (Figure 4c). Compared with pure SnS nanoparticles and SnS/G, the N-doped graphene sheets offered more active sites for SnS, and thus could more effectively resist the aggregation of SnS nanoparticles during the preparation process. 

**Figure 5:**
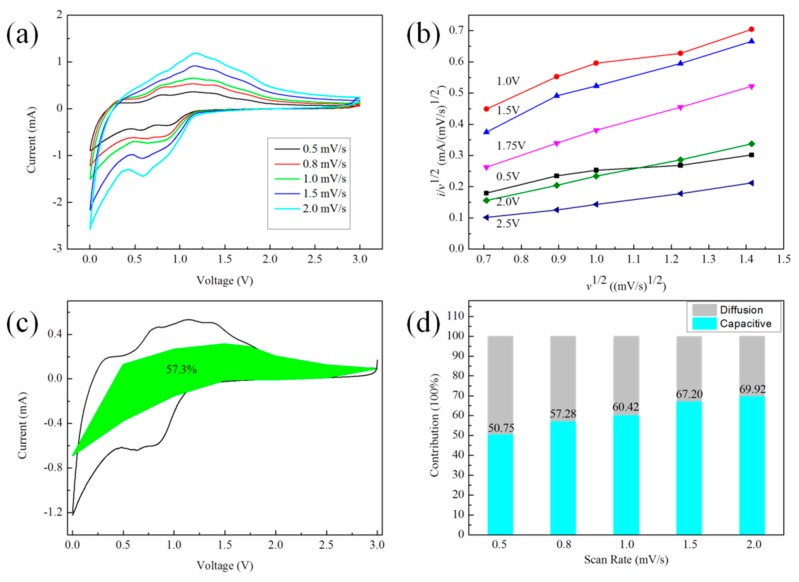
Quantitative capacitive analysis of sodium storage behavior of SnS/NG. (**a**) Cyclic voltammetry (CV) curves at different scan rates. (**b**) Plots of *v*^1/2^ vs. *i*/*ν*^1/2^. (**c**) Capacitive contribution at 0.8 mV/s. (**d**) Normalized contribution ratio of capacitive capacities at different scan rates.

Figure 5a shows the cyclic voltammetry (CV) tests at different scan rates, which was used to better understand the fast sodium storage kinetics of SnS/NG. The shape is well preserved, with increasing scan rate from 0.5 to 2.0 mV/s. The peak at 0.6V corresponds to alloying and conversion reaction kinetics that form the Na_15_S_4_ phase [6]. The peak at 1.17 V refers to the reformation of SnS. In CV measurements, the total current measured under a potential sweep rate may be interpreted as the sum of the current in relation to the slow diffusion-controlled process (idiff) and the current required to charge a double layer at the electrolyte interface or to initiate fast faradaic reactions on an exposed electrode surface (icap) [13]. Thus, quantitatively distinguishing between capacitive processes and diffusion-controlled intercalation processes is highly desirable for a better understanding of the underlying charge storage mechanism, which aids in the selection of materials and device design [14].

The relationship between *i*(V)and *v*^1/2^ may be described using the following equation (1):

(1) $$\frac{i\left(V\right)}{v^{\frac{1}{2}}}=k_{1}v^{\frac{1}{2}}+k_{2}$$

The constants *k*_1_ and *k*_2_ can be evaluated from the slope and intercept, respectively, of a linear plot of *i*(V)/*v*^1/2^ versus *v*^1/2^. Consequently, it is possible to quantitatively differentiate the current contribution from the capacitive effect (*k*_1_*v*) from diffusion-controlled intercalation processes (*k*_2_*v*^1/2^) (Figure 5b) [14].

In Figure 5c, the percentage of capacitive contribution to the current at a fixed voltage may be quantitatively determined by separating the current response, *i*, from the diffusion-controlled and capacitive contribution at the corresponding voltage [15,16]. As a result, 57.3% of the total capacity was identified as the capacitive contribution at 0.8 mV/s. With an increase in scan rate, the diffusion contribution depressed, while the capacitive contribution—as expected—increased (Figure 5d). At 2.0 mV/s, the capacitive contribution tended to be stable at 69.92%. Thus, for the fast sodium storage kinetics of SnS/NG, the capacity was mainly drawn from the capacitive contribution. 

**Figure 6:**
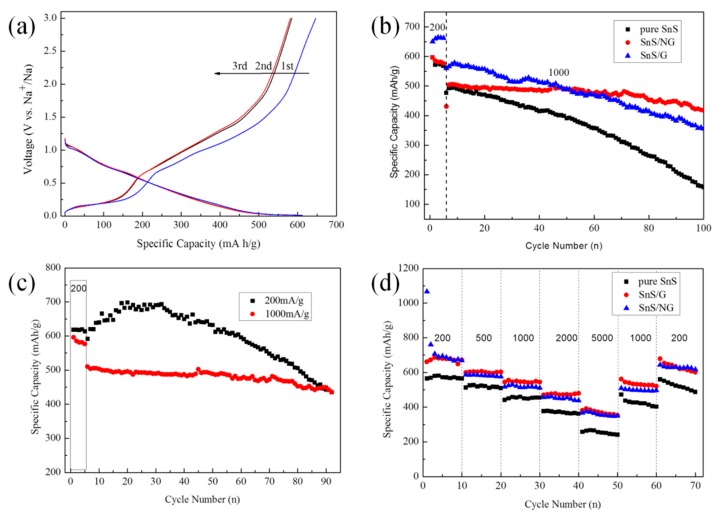
(**a**) Discharge/charge profiles of SnS/NG at different cycles at 200 mA/g. (**b**) Cycle stability of SnS/NG, SnS/G and SnS at 1000 mA/g. (**c**) Cycle stability of SnS/NG at 1000 mA/g and 200 mA/g. (**d**) Rate performance of SnS/NG, SnS/G and SnS.

The initial discharge/charge profiles of SnS/NG at 200 mA/g are shown in Figure 6a. The discharge capacity and charge capacity at the first cycle were found to be 612.8 and 647.1 mA h/g, respectively. The electrode showed good reversibility after a stable solid electrolyte interphase (SEI) had been formed. For the second and third cycle, the voltage profiles overlapped, while the coulombic efficiency was determined to be 100%. At the current density of 1000 mA/g, the long-time cycle stability of SnS, SnS/G and SnS/NG are shown in Figure 6b. In the sixth cycle, the specific capacity of SnS/NG, SnS/G and SnS were 504.0, 566.3 and 492.1 mAh/g, respectively, while after 100 cycles, the specific capacity of SnS/NG, SnS/G and SnS were 417.8, 355.6 and 157.9 mAh/g, respectively. We ascribed the rapid decline of the capacity for SnS to low electrical conductivity of SnS and severe aggregation during the discharge/charge cycles. Compared with SnS/G, SnS/NG exhibited better cycle performance. Obviously, the N-doped graphene sheets had an important role in promoting the cycle stability of SnS/NG. In order to verify the influence of current density on the cycle stability of SnS/NG, 200 mA/g and 1000 mA/g were selected. In the first cycle, the capacity of pure SnS and SnS/NG were 596.65 mAh/g and 596.34 mAh/g, respectively, with the capacity contribution of N-doped graphene to the composite determined as 180.77 mAh/g, which was calculated using:

(2) $${\mathrm{Capacity}}_{\mathrm{NG}}={\mathrm{Capacity}}_{\mathrm{SnS}/\mathrm{NG}}-{\mathrm{Content}}_{\mathrm{SnS}}\cdot {\mathrm{Capacity}}_{\mathrm{SnS}}=596.34\;\mathrm{mAh}/g-69.95\%\times 596.65\;\mathrm{mA}/g=180.77\;\mathrm{mAh}/g$$

As shown in Figure 6c, the cycle stability of SnS/NG at 1000 mA/g was better than that at 200 mA/g. After 100 cycles, the capacity retention of SnS/NG at 1000 mA/g and 200 mA/g were 80.1% and 57.4%, respectively. Thus, at a higher current density, SnS/NG had better cycling stability, indicating strong potential for its use in high-power SIBs. Figure 6d shows the rate performance of SnS/NG, SnS/G and SnS, in which the current densities increase step-wise from 200 mA/g to 5000 mA/g, and finally back to 200 mA/g. During the process, SnS/NG and SnS/G consistently outperformed SnS. In the first 10 cycles, the specific capacity of SnS/NG was 683.8 mAh/g at 200 mA/g, while at a current density of 5000 mA/g, a specific capacity of 366.9 mAh/g was maintained. When the current density returned to 1000 and 200 mA/g, the discharge capacities were 499.3 and 628.4 mAh/g, respectively, which were only marginally lower than those initial values at the same current densities. The superior rate capability could be attributed to the intricate structure of SnS/NG, which enables it to withstand a harsh environment. 

**Figure 7:**
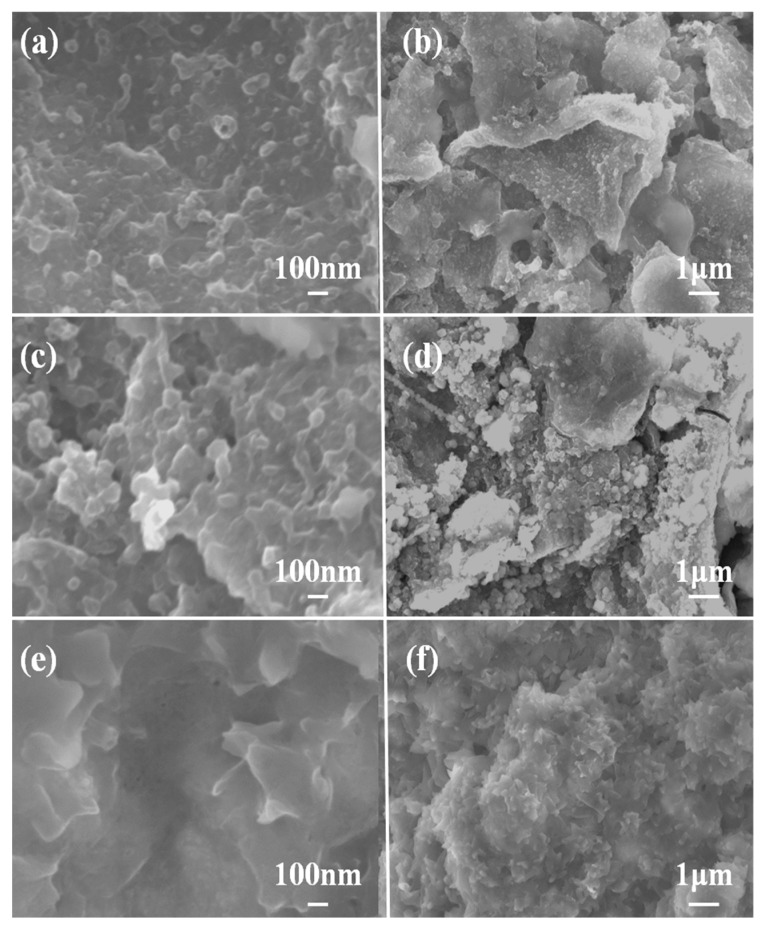
SEM images of (**a**,**b**) SnS/NG, (**c**,**d**) SnS/G, and (**e**,**f**) SnS after 100 cycles.

To further understand the superior cycle life of the SnS/NG anode, the morphologies of the samples after 100 cycles at 1000 mA g^−1^ were further investigated. As shown in Figure 7, SnS/NG electrodes maintained, for the most part, their structural integrity after 100 cycles, while SnS and SnS/G electrodes had obvious structural failure. Additionally, the cracks of the SnS electrodes were more severe than either the SnS/NG or SnS/G electrodes. For the cycled SnS/G hybrid, SnS clusters situated outside the graphene layers were visible due to a significant detachment from graphene sheets and subsequent aggregation into larger particles. In comparison, most of the SnS in SnS/NG were still well-dispersed, suggesting a strong attraction between them. 

In order to further test the cycle performance of the SnS/NG obtained in our work as a potential anode material in sodium ion batteries, we compared the electrochemical performances of various SnS/graphene materials. The materials compared are listed in Table 1. Through contrast, we found that the cycle performance of the SnS/NG obtained in our work at high current density is excellent. Thus, our simple and gentle synthesis method may be used for reference in the preparation of other materials.

## 4. Conclusions

In summary, SnS anchored to NG sheets was prepared in-situ using a facile, gentle, two-step process combining a hot water bath reaction and thermal annealing. Owing to its structural advantages, SnS/NG exhibited good cycling stability and excellent rate capability in comparison to pure SnS. With 35.35% content of graphene in the SnS/NG composite, the specific capacity was found to still be 417.8 mAh/g at 1000 mA/g after 100 cycles, which reveals a high specific capacity and cycling stability. These excellent electrochemical properties demonstrate SnS may be applied in high-performance SIB fields. Furthermore, we analyzed the fast sodium storage kinetics of SnS/NG, providing some theoretical support for further study.

## Figures and Tables

**Table 1:** Comparison of cycling performance of SnS/NG with previously reported Sn-based anodes for sodium ion batteries (SIBs).

Materials	Synthesis Method	Reversible Capacity (mAh/g)	Cycle Life	Current Density (mA/g)	Carbon Content (%)	Ref.
SnS nanoparticles /reduced graphene oxide	In-situ growing + annealing (700 °C)	559	70	200	31.4	[5]
SnS@RGO	Precipitation + annealing (400 °C)	386	100	100	21.9	[17]
SnS/G	Hot water bath + annealing (650 °C)	<300	30	50	15	[12]
hN-C@SnS	Microwave reaction + annealing	441.8	100	200	28.7	[18]
SnS/NG	Hot water bath + annealing (600 °C)	417.8	100	1000	30.35	In our work
